# The Impacts of COVID-19 on Technology Use and Experience Across Generations

**DOI:** 10.1093/geroni/igab046.311

**Published:** 2021-12-17

**Authors:** Chaiwoo Lee, Shabnam FakhrHosseini, John Rudnik

**Affiliations:** 1 Massachusetts Institute of Technology, Cambridge, Massachusetts, United States; 2 MIT AgeLab, Cambridge, Massachusetts, United States; 3 University of Michigan School of Information

## Abstract

Among its many downstream effects, the COVID-19 pandemic has influenced how people think about and interact with technology. With limitations and restrictions around in-person interactions and use of public spaces, people are increasingly relying on technology to support everyday activities including work, communication and care. Results from the survey series showed an increased adoption of and interest in home, health and communication technologies. The changes may be long-lived, with the majority of the new adopters saying that they will continue to use the technologies that they started using in response to the pandemic. A generational comparison showed that while baby boomers and the silent generation were less likely than younger adults to have made recent adoptions, the older generations did not significantly differ in terms of interest in using new technologies. This presentation will also report on how COVID-19-related changes in technology experience varied by other demographic and socio-economic characteristics.

